# Health Problems of Company Employees With Cancer in Japan: A Cross-Sectional Study

**DOI:** 10.7759/cureus.81502

**Published:** 2025-03-31

**Authors:** Shuko Hotoge, Mieko Abe, Takafumi Monma, Sakiko Ozawa, Fumi Takeda

**Affiliations:** 1 Institute of Health and Sport Sciences, University of Tsukuba, Tsukuba, JPN

**Keywords:** breast cancer, cervical cancer, employees, gastrointestinal cancer, health problems

## Abstract

Background: With an increase in employees with cancer, employers are making efforts to ensure that employees with cancer can continue to work while maintaining and improving their physical and mental health, just as those without cancer. However, little is known about how the health conditions of employees undergoing cancer treatment differ from those of employees without cancer. This study aimed to examine health problems other than cancer in employees with cancer compared to those without cancer, stratified based on sex and cancer type.

Methods: This study was conducted using data from the Stress Check Program and health insurance claims for Japanese corporate employees in 2015 and 2016. Stratified based on sex and cancer type, 19 health problems such as depressive symptoms, anxiety, indefinite complaints, and diseases in 219 employees with cancer (56 males with gastrointestinal cancer, 96 females with cervical cancer, and 67 females with breast cancer) and 14,017 employees without cancer were compared. Logistic regression analyses were performed with each health problem as the objective variable, cancer as the explanatory variable, and age, job position, and department as the adjusted variables.

Results: Male employees with gastrointestinal cancer had lower odds ratios (ORs) for eyestrain and lower back pain than those without cancer. Female employees with cervical cancer had higher ORs for heart diseases and hypertension, and those with breast cancer had higher ORs for musculoskeletal diseases than those without cancer.

Conclusions: Male employees with gastrointestinal cancer had less eyestrain and lower back pain than those without cancer. Female employees with cervical cancer had more heart diseases and hypertension than those without cancer, whereas those with breast cancer had more musculoskeletal diseases than those without cancer. Therefore, workplace support measures for these health problems among female employees with cervical or breast cancer are needed.

## Introduction

In Japan, approximately one million people are newly diagnosed with cancer yearly, and more than 30% of them are aged between 15 and 64 years (the working age) [1]. The number of workers under cancer treatment was 499,000 in 2022, an increase of more than 170,000 from that in 2010 [2]. With an increase in employees with cancer, support for creating a balance between treatment and work has been enhanced through employment measures that ensure time for medical visits and cancer treatment, as well as guidelines for developing a working environment that considers the side effects of cancer treatment [2,3]. Employers are currently making various efforts to ensure that employees with cancer can continue to work while maintaining and improving their physical and mental health, just as those without cancer.

However, little is known about how the health conditions of employees undergoing cancer treatment differ from those of employees without cancer. To date, few previous studies have examined depressive symptoms, anxiety, and indefinite complaints in workers who had received treatment in the past or had completed cancer treatment; however, their findings were inconsistent. For example, a study of U.S. female workers reported that those who had a history of primary breast cancer with completed treatment had stronger depressive symptoms and anxiety than those who did not have cancer, adjusting for age [4]. Conversely, a study of Japanese workers reported that depressive symptoms in those who had been treated for stomach, colorectal, lung, liver, breast, prostate, or other cancers did not differ from those without cancer in both sexes, adjusting for age, employment type, and social, physical, and psychological characteristics [5]. Another study of workers in Denmark, Finland, Iceland, and Norway reported that depressive symptoms in those who were tumor-free after primary treatment for breast, prostate, or testicular cancer diagnosed two to six years prior did not differ from those without cancer, adjusting for sex. However, anxiety and overall indefinite complaints were higher in these workers than in those without cancer, adjusting for sex [6]. In addition, the latter two studies pointed out that these health problems of workers had been treated or after primary treatment for cancer might differ according to the cancer type [5,6].

Furthermore, to our knowledge, no studies have examined diseases other than cancer in workers with cancer. A nationwide study in the U.S. reported that adults with cancer have a higher prevalence of hypertension, coronary artery disease, dyslipidemia, diabetes, and arthritis than those without cancer [7]; however, the study did not consider sex, age, or job attributes. It is possible that the status of such diseases in workers with cancer differs from those without cancer.

In Japan, employers have been mandated to undergo stress checks for the early detection and treatment of poor mental health and indefinite complaints of employees [8]. Therefore, the data of the Stress Check Program and insurance claims are the most reliable real-world data for mental health, indefinite complaints, and various diseases in Japanese employees. It is useful to examine the health problems of employees with cancer compared to those without cancer by using these data.

Therefore, this study aimed to identify health problems specific to employees with cancer stratified based on sex and cancer type, by comparison to those without cancer in terms of depressive symptoms, anxiety, indefinite complaints, and diseases other than cancer, using data from the Stress Check Program and health insurance claims in Japan. This study’s findings would provide empirical evidence on the health problems of Japanese employees with cancer based on real-world data and contribute to the examination of effective health support measures.

## Materials and methods

Materials and procedures

For this cross-sectional study, purposive sampling was used to select company employees with both stress check and health insurance claim data. We used anonymous, complete Stress Check Program and health insurance claims data for fiscal years 2015 and 2016 for 14,549 employees aged 21-69 belonging to a Japanese company, which were provided for academic use under the non-disclosure agreement of the company and health insurance association.

**Figure 1:**
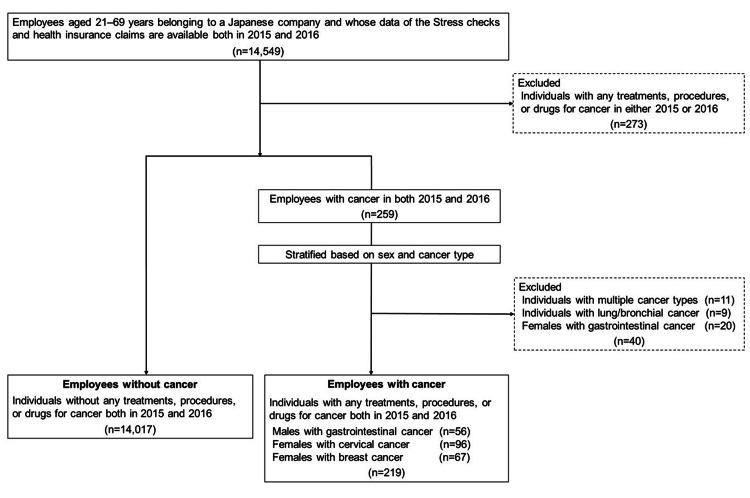
Flowchart of analysis data

The flow chart for the selection of subjects for analysis is shown in Figure 1. First, we matched health insurance claims from 2015 and 2016 for 14,549 company employees who underwent a stress check in 2016 (conducted in July and August) to extract 259 people who had received cancer treatments, procedures, or drugs in both years, which we defined as "employees with cancer," and 14,017 people who had not received cancer treatment, procedures, or drugs in either year, which we defined as "employees without cancer." The reason for matching health insurance claims for the two years is that the data used in the analysis is a 12-month total, and the month of the visit to the healthcare provider is unknown, so we extracted employees who had received cancer treatment, procedures, or drugs before the 2016 stress check.

The cancer type corresponded with the International Statistical Classification of Diseases and Related Health Problems version 10 codes (ICD-10) [9] and is as follows: oral/pharyngeal/laryngeal cancer (C00-C14 and C32), esophageal cancer (C15), stomach cancer (C16), colon cancer (C18-C20), biliary/liver/pancreatic cancer (C22-C25), lung/bronchial cancer (C33, C34), breast cancer (C50), and cervical cancer (C53). We converted the cancer types into four groups as follows: "gastrointestinal cancer" (including oral/pharyngeal/laryngeal cancer (C00-C14 and C32), esophageal cancer (C15), stomach cancer (C16), colon cancer (C18-C20), biliary/liver/pancreatic cancer (C22-C25)), "lung/bronchial cancer (C33, C34)," "breast cancer (C50)," and "cervical cancer (C53)."

Then, we calculated the sample size required for analysis of employees with cancer stratified by sex and type of cancer based on the criteria of a two-tailed test, α error = 0.05, power = 0.8, and effect size (Medium: t-test = 0.5, Mann-Whitney U test = 0.5, chi-square test = 0.3). The results were 64 for the t-test, 67 for the Mann-Whitney U test, and a total of 143 for the chi-square test. Based on this, nine cases of lung/bronchial cancer and 20 cases of female gastrointestinal cancer were excluded from the analysis due to their small numbers. In addition, 11 cases of multiple cancer types were excluded, and a total of 219 cases were ultimately analyzed: 96 females with cervical cancer, 67 females with breast cancer, and 56 males with gastrointestinal cancer (Figure 1). The reliability of the statistical tests is sufficient for 96 females with cervical cancer and 67 females with breast cancer, but may be slightly inferior for 56 males with gastrointestinal cancer in the t-test and Mann-Whitney U test.

This study was approved by the ethics committee of the Institute of Health and Sport Sciences, University of Tsukuba, Tsukuba, Japan, in compliance with the Declaration of Helsinki (reference number: Tai 29-132). There was no informed consent because the anonymous data were provided by the company and the health insurance association.

Measures

Attributes

Attributes including age, sex, job position (manager or non-manager), and department (sales, customer service, or administration) were obtained.

Health Problems

Based on previous studies [4-7], we used 19 health problems from the data of the stress checks, which are self-administered questionnaires established by the Ministry of Health, Labour, and Welfare in Japan [10, 11], and of health insurance claims in 2016.

Regarding depressive symptoms and anxiety, respondents were asked to answer nine questions in the stress checks, comprising six items for depressive symptoms and three for anxiety, using a four-point scale, with four indicating almost always, three indicating often, two indicating sometimes, and one indicating almost never. Each total score of depressive symptoms and anxiety was converted into a high or low condition based on the Stress Check Score Conversion Chart [11] as follows: depressive symptoms, a total score of ≥ 13 indicated "high depressive symptoms," whereas a score of ≤ 12 indicated "low depressive symptoms," anxiety, a total score of ≥ eight indicated "high anxiety," whereas a score of ≤ seven indicated "low anxiety."

Regarding the 11 indefinite complaints (dizziness, joint pains, headaches, a stiff neck and/or shoulders, lower back pain, eyestrain, heart palpitations or shortness of breath, stomach and/or intestine problems, lack of appetite, diarrhea and/or constipation, and insufficient sleep), respondents were asked to answer the following questions in stress checks: "I have felt dizzy," "I have experienced joint pains," "I have experienced headaches," "I have had a stiff neck and/or shoulders," "I have had lower back pain." "I have had eyestrain." "I have experienced heart palpitations or shortness of breath," "I have experienced stomach and/or intestine problems," "I have lost my appetite," "I have experienced diarrhea and/or constipation," and "I have not been able to sleep well." In each item, an answer of "almost never" was considered a "no," and answers of "sometimes," "often," and "almost always" were considered a "yes."

Six diseases (hypertension, heart diseases (except hypertension), dyslipidemia, diabetes, musculoskeletal diseases, and mental illness) were assessed from the health insurance claims data, including treatments, procedures in the hospital, drugs, and duration for disease. We determined the presence of diseases based on whether individuals received any treatments, procedures, or drugs for the primary diseases, regardless of the duration. The six disease items correspond to the ICD-10 codes [9] as follows: hypertension (I10-I15); heart diseases (except hypertension) (I01-I02.0, I05-I09, I20-I25, I27, I30-I52); dyslipidemia (E78); diabetes (E10-E14); musculoskeletal diseases (M00-M99); mental illness (mood (affective) disorders (F30-F39), neurotic, stress-related and somatoform disorders (F40-F48), other mental and behavioral disorders (except for dementia (F01, F03), mental and behavioral disorders due to psychoactive substance use (F10-F19), schizophrenia (F20-F29), and mental retardation (F70-F79)), and disorders of the autonomic nervous system (G90)).

Statistical analysis

After observing the basic statistics of all variables, we compared attributes and health problems of those with and without cancer using the t-test, chi-square, or Fisher’s exact tests. Subsequently, 19 logistic regression analyses were conducted based on sex and cancer type, with each health problem as the objective variable (depressive symptoms and anxiety (low: 0; high: 1), 11 indefinite complaints, and six diseases (no: 0; yes: 1)), cancer as the explanatory variable (absence: 0, presence: 1; gastrointestinal cancer for males; cervical and breast cancer for females), and age, job position, and department as the adjusted variables. In both univariate and multivariate analyses, P-value correction using the Benjamini-Hochberg method was conducted to consider multiple comparisons problems. This is because the Benjamini-Hochberg method controls the false discovery rate and tolerates some errors, and is used in exploratory analysis where many rejection hypotheses are desired. In contrast, other methods, such as the Bonferroni and Holm methods, control the family-wise error rate and have low power due to strict significance level settings and are used, for example, in pharmaceutical validation analyses. All data analyses were performed using IBM SPSS Statistics software, version 25.0 for Windows (IBM Corp., Armonk, NY, USA). Statistical significance was set at p < 0.05.

## Results

Results

The study population’s characteristics of attributes, presence of cancer, and health problems are shown in Table 1. A summary of the attributes and health problems of employees with and without cancer is shown in Table 2. Of these (6,732 males and 7,504 females), the employees with cancer were 56 males (gastrointestinal cancer) and 163 females (96 with cervical cancer and 67 with breast cancer), respectively.

**Table 1:** Subject characteristics SD: standard deviation a: P-value correction using the Benjamini-Hochberg method was conducted to consider multiple comparisons problems; b: T-test; c: Chi-square test; d: Fisher's exact test; e: Percentages were calculated using the total number of those with cancer as the population

Variables		All	Male	Female	P-value^a^
		n = 14236	n = 6732	n = 7504	
		Mean ± SD or n (%)	Mean ± SD or n (%)	Mean ± SD or n (%)	
Age	Overall	43.0 ± 11.9	47.9 ± 11.1	38.5 ± 10.8	<0.001^b^
	21–29 years	2464 (17.3)	480 (7.1)	1984 (26.4)	<0.001^c^
	30–39 years	3421 (24.0)	1139 (16.9)	2282 (30.5)	
	40–49 years	3667 (25.8)	1932 (28.7)	1735 (23.1)	
	50–59 years	3347 (23.5)	2032 (30.2)	1315 (17.5)	
	60–69 years	1337 (9.4)	1149 (17.1)	188 (2.5)	
Job position	Manager	2350 (16.5)	2191 (32.5)	159 (2.1)	<0.001^c^
	Non-manager	11886 (83.5)	4541 (67.5)	7345 (97.9)	
Department	Sales	7612 (53.5)	3137 (46.6)	4475 (59.6)	<0.001^c^
	Customer service	4557 (32.0)	2444 (36.3)	2113 (28.2)	
	Administration	2067 (14.5)	1151 (17.1)	916 (12.2)	
Cancer	Absence	14017 (98.5)	6676 (99.2)	7341 (97.8)	<0.001^d^
	Presence	219 (1.5)	56 (0.8)	163 (2.2)	
	Gastrointestinal cancer^e^	56 (25.6)	56 (100.0)	0 (0.0)	-
	Cervical cancer^e^	96 (43.8)	0 (0.0)	96 (58.9)	
	Breast cancer^e^	67 (30.6)	0 (0.0)	67 (41.1)	
Depressive symptoms	High	2775 (19.5)	847 (12.6)	1928 (25.7)	<0.001^c^
Anxiety	High	3517 (24.7)	1316 (19.5)	2201 (29.3)	<0.001^c^
Dizziness	Yes	3816 (26.8)	1099 (16.3)	2717 (36.2)	<0.001^c^
Joint pains	Yes	3972 (27.9)	1603 (23.8)	2369 (31.6)	<0.001^c^
Headaches	Yes	6923 (48.6)	2157 (32.0)	4766 (63.5)	<0.001^c^
A stiff neck and/or shoulders	Yes	11369 (79.9)	4663 (69.3)	6706 (89.4)	<0.001^c^
Lower back pain	Yes	8313 (58.4)	3559 (52.9)	4754 (63.4)	<0.001^c^
Eyestrain	Yes	12617 (88.6)	5634 (83.7)	6983 (93.1)	<0.001^c^
Heart palpitations or shortness of breath	Yes	3307 (23.2)	1258 (18.7)	2049 (27.3)	<0.001^c^
Stomach and/or intestine problems	Yes	6561 (46.1)	2571 (38.2)	3990 (53.2)	<0.001^c^
Lack of appetite	Yes	4063 (28.5)	1420 (21.1)	2643 (35.2)	<0.001^c^
Diarrhea and/or constipation	Yes	6736 (47.3)	2746 (40.8)	3990 (53.2)	<0.001^c^
Insufficient sleep	Yes	6377 (44.8)	2760 (41.0)	3617 (48.2)	<0.001^c^
Hypertension	Yes	1661 (11.7)	1331 (19.8)	330 (4.4)	<0.001^c^
Heart diseases	Yes	595 (4.2)	425 (6.3)	170 (2.3)	<0.001^c^
Dyslipidemia	Yes	3444 (24.2)	1801 (26.8)	1643 (21.9)	<0.001^c^
Diabetes	Yes	882 (6.2)	691 (10.3)	191 (2.5)	<0.001^c^
Musculoskeletal diseases	Yes	3559 (25.0)	1793 (26.6)	1766 (23.5)	<0.001^c^
Mental illness	Yes	1029 (7.2)	469 (7.0)	560 (7.5)	1.000^c^

**Table 2:** Attributes and health problems of employees with and without cancer SD: standard deviation a: P-value correction using the Benjamini-Hochberg method was conducted to consider multiple comparisons problems; b: T-test; c: Fisher’s exact test

Variables		Male			Female				
		Without cancer	Gastrointestinal cancer	P-value^a^	Without cancer (i)	Cervical cancer (ii)	(i) vs (ii)	Breast cancer (iii)	(i) vs (ⅲ)
		n = 6676	n = 56		n = 7341	n = 96		n = 67	
		Mean ± SD or n (%)	Mean ± SD or n (%)		Mean ± SD or n (%)	Mean ± SD or n (%)	P-value^a^	Mean ± SD or n (%)	P-value^a^
Age	Overall	47.8 ± 11.1	58.9 ± 5.0	<0.001^b^	38.4 ± 10.8	39.4 ± 9.2	0.314^b^	50.2 ± 6.8	<0.001^b^
	21–29 years	480 (7.2)	0 (0.0)	<0.001^c^	1969 (26.8)	14 (14.6)	0.020^c^	1 (1.5)	<0.001^c^
	30–39 years	1139 (17.1)	0 (0.0)		2242 (30.6)	37 (38.6)		3 (4.5)	
	40–49 years	1929 (28.9)	3 (5.4)		1684 (22.9)	25 (26.0)		26 (38.8)	
	50–59 years	2008 (30.0)	24 (42.9)		1262 (17.2)	20 (20.8)		33 (49.2)	
	60–69 years	1120 (16.8)	29 (51.7)		184 (2.5)	0 (0.0)		4 (6.0)	
Job position	Manager	2178(32.6)	13 (23.2)	0.320^c^	153 (2.1)	1 (1.0)	1.000^c^	5 (7.5)	0.017^c^
	Non-manager	4498 (67.4)	43 (76.8)		7188 (97.9)	95 (99.0)		62 (92.5)	
Department	Sales	3120 (46.7)	17 (30.4)	0.064^c^	4369 (59.5)	62 (64.5)	0.210^c^	44 (65.6)	1.000^c^
	Customer service	2417 (36.2)	27 (48.2)		2067 (28.2)	28 (29.2)		18 (26.9)	
	Administration	1139 (17.1)	12 (21.4)		905 (12.3)	6 (6.3)		5 (7.5)	
Depressive symptoms	High	842 (12.6)	5 (8.9)	1.000^c^	1893 (25.8)	22 (22.9)	1.000^c^	13 (19.4)	0.433^c^
Anxiety	High	1312 (19.7)	4 (7.1)	0.024^c^	2158 (29.4)	26 (27.1)	1.000^c^	17 (25.4)	1.000^c^
Dizziness	Yes	1094 (16.4)	5 (8.9)	0.285^c^	2657 (36.2)	40 (41.7)	0.365^c^	20 (29.9)	0.546^c^
Joint pains	Yes	1592 (23.8)	11 (19.6)	1.000^c^	2307 (31.4)	30 (31.3)	1.000^c^	32 (47.8)	0.006^c^
Headaches	Yes	2144 (32.1)	13 (23.2)	0.357^c^	4658 (63.5)	63 (65.6)	1.000^c^	45 (67.2)	1.000^c^
A stiff neck and/or shoulders	Yes	4631 (69.4)	32 (57.1)	0.079^c^	6556 (89.3)	89 (92.7)	0.661^c^	61 (91.0)	1.000^c^
Lower back pain	Yes	3537 (53.0)	22 (39.3)	0.059^c^	4646 (63.3)	64 (66.7)	1.000^c^	44 (65.7)	1.000^c^
Eyestrain	Yes	5592 (83.8)	42 (75.0)	0.136^c^	6829 (93.0)	91 (94.8)	1.000^c^	63 (94.0)	1.000^c^
Heart palpitations or shortness of breath	Yes	1251 (18.7)	7 (12.5)	0.670^c^	2001 (27.3)	27 (28.1)	1.000^c^	21 (31.3)	1.000^c^
Stomach and/or intestine problems	Yes	2550 (38.2)	21 (37.5)	1.000^c^	3905 (53.2)	48 (50.0)	1.000^c^	37 (55.2)	1.000^c^
Lack of appetite	Yes	1406 (21.1)	14 (25.0)	1.000^c^	2589 (35.3)	35 (36.5)	1.000^c^	19 (28.4)	0.385^c^
Diarrhea and/or constipation	Yes	2720 (40.7)	26 (46.4)	1.000^c^	3894 (53.0)	56 (58.3)	0.479^c^	40 (59.7)	0.624^c^
Insufficient sleep	Yes	2740 (41.0)	20 (35.7)	1.000^c^	3530 (48.1)	51 (53.1)	0.511^c^	36 (53.7)	0.817^c^
Hypertension	Yes	1312 (19.7)	19 (33.9)	0.009^c^	315 (4.3)	8 (8.3)	0.077^c^	7 (10.4)	0.033^c^
Heart diseases	Yes	419 (6.3)	6 (10.7)	0.444^c^	161 (2.2)	7 (7.3)	0.006^c^	2 (3.0)	1.000^c^
Dyslipidemia	Yes	1779 (26.6)	22 (39.3)	0.045^c^	1598 (21.8)	24 (25.0)	0.806^c^	21 (31.3)	0.105^c^
Diabetes	Yes	676 (10.1)	15 (26.8)	0.007^c^	187 (2.5)	2 (2.1)	1.000^c^	2 (3.0)	1.000^c^
Musculoskeletal diseases	Yes	1769 (26.5)	24 (42.9)	<0.001^c^	1710 (23.3)	26 (27.1)	0.606^c^	30 (44.8)	<0.001^c^
Mental illness	Yes	467 (7.0)	2 (3.6)	1.000^c^	543 (7.4)	7 (7.3)	1.000^c^	10 (14.9)	0.042^c^

Male employees with gastrointestinal cancer had a higher mean age (58.9 ± 5.0 years) than those without cancer (47.8 ± 11.1 years), and 94.6% of them were aged ≥ 50 years. They had a higher percentage of those with hypertension, dyslipidemia, diabetes, and musculoskeletal diseases than those without cancer. Female employees with cervical cancer had a higher percentage of heart diseases than those without cancer. Female employees with breast cancer had a higher mean age (50.2 ± 6.8 years) than those without cancer (38.4 ± 10.8 years), and 94.0% of them were aged ≥ 40 years. They had a higher proportion of managers and a higher percentage of those with joint pains, hypertension, musculoskeletal diseases, and mental illness than those without cancer.

**Figure 2:**
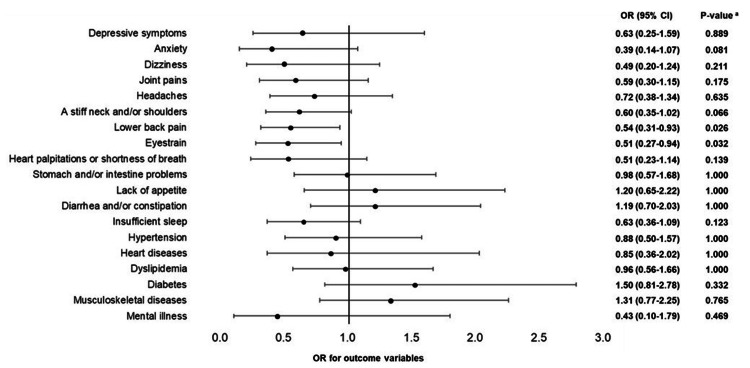
Health problems of males with gastrointestinal cancer (logistic regression analysis) Nineteen logistic regression analyses were conducted, 19 of which were objective variables (depressive symptoms and anxiety (low: 0; high: 1), 11 indefinite complaints, and six diseases (no: 0; yes: 1)). Cancer was the explanatory variable (reference variable (0) was "absence"). Age, job position, and department were adjusted variables. OR: odds ratio; Cl: confidence interval; a: P-value correction using the Benjamini-Hochberg method was conducted to consider multiple comparisons problems.

**Figure 3:**
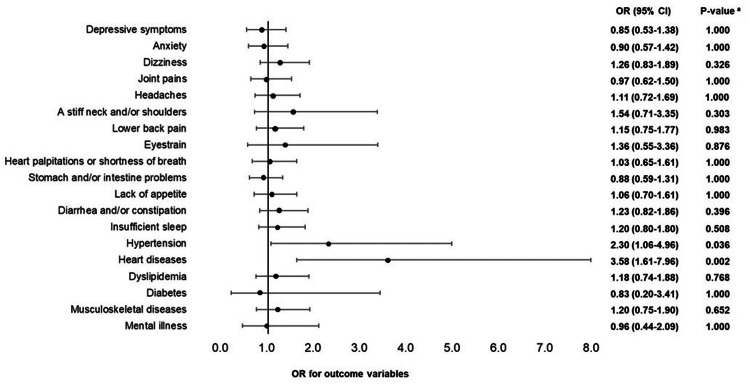
Health problems of females with cervical cancer (logistic regression analysis) Nineteen logistic regression analyses were conducted, 19 of which were objective variables (depressive symptoms and anxiety (low: 0; high: 1), 11 indefinite complaints, and six diseases (no: 0; yes: 1)). Cancer was the explanatory variable (reference variable (0) was "absence"). Age, job position, and department were adjusted variables. OR: odds ratio; Cl: confidence interval; a: P-value correction using the Benjamini-Hochberg method was conducted to consider multiple comparisons problems.

**Figure 4:**
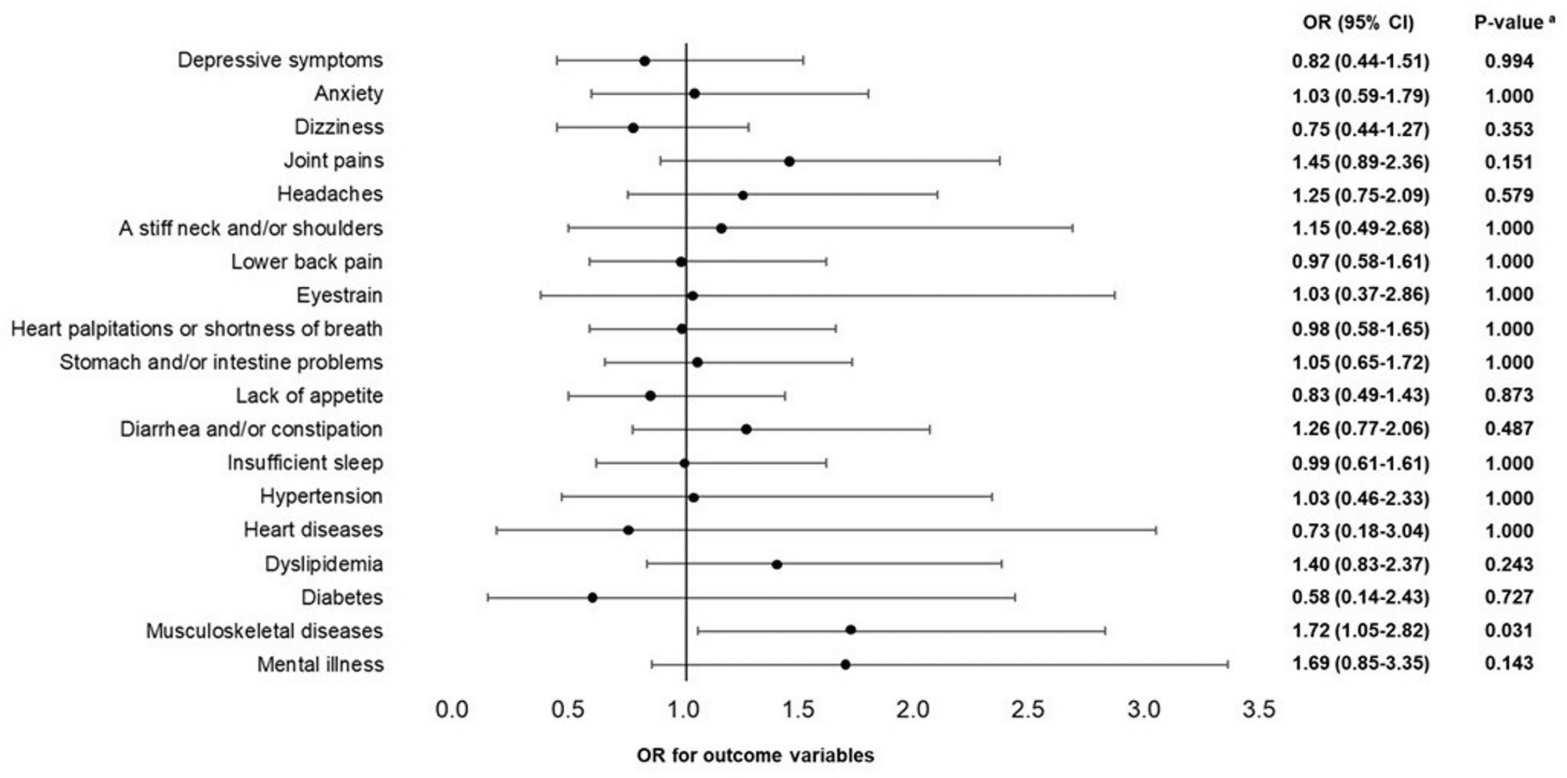
Health problems of females with breast cancer (logistic regression analysis) Nineteen logistic regression analyses were conducted, 19 of which were objective variables (depressive symptoms and anxiety (low: 0; high: 1), 11 indefinite complaints, and six diseases (no: 0; yes: 1)). Cancer was the explanatory variable (reference variable (0) was "absence"). Age, job position, and department were adjusted variables. OR: odds ratio; Cl: confidence interval; a: P-value correction using the Benjamini-Hochberg method was conducted to consider multiple comparisons problems.

The results of the logistic regression analyses adjusted for age, job position, and department for males with gastrointestinal cancer, females with cervical cancer, and females with breast cancer are shown in Figures 2-4, respectively. In males, employees with gastrointestinal cancer had lower odds ratios (ORs) of "eyestrain" (OR = 0.51, 95% confidence interval (Cl) = 0.27-0.94, adjusted p = 0.032) and "lower back pain" (OR = 0.54, 95% Cl = 0.31-0.93, adjusted p = 0.026) than those without cancer. In females, employees with cervical cancer had higher ORs of "heart diseases" (OR = 3.58, 95% CI = 1.61-7.96, adjusted p = 0.002) and "hypertension" (OR = 2.30, 95% CI = 1.06-4.96, adjusted p = 0.036), whereas employees with breast cancer had a higher OR of "musculoskeletal diseases" (OR = 1.72, 95% CI = 1.05-2.82, adjusted p = 0.031) than those without cancer.

## Discussion

This study examined health problems such as depressive symptoms, anxiety, indefinite complaints, and diseases other than cancer in employees with cancer compared to those without cancer, stratified based on sex and cancer type. The age distribution of those with cancer in this study population was consistent with the preferred age of incidence for each cancer (gastrointestinal cancer: age ≥ 40 years, cervical cancer: age 30-40 years, and breast cancer: age 40-60 years [12]).

The key results of this study are that male employees with gastrointestinal cancer had less eyestrain and lower back pain than those without cancer. On the other hand, female employees with cervical cancer had more heart diseases and hypertension, whereas those with breast cancer had more musculoskeletal diseases, respectively, than those without cancer.

First, male employees with gastrointestinal cancer experienced lower ORs for eyestrain and lower back pain than those without cancer. Although male employees with gastrointestinal cancer had a higher rate of hypertension, dyslipidemia, diabetes, and musculoskeletal diseases than those without cancer (Table 2), which are consistent with reports on adults with cancer in the U.S. [7], these differences disappeared after adjusting for age, job position, and department (Figure 2). Our findings suggest that male employees with gastrointestinal cancer may have a lower risk of eyestrain and lower back pain than those without cancer. According to a previous study that examined the work environment in the same population as the subject of this study, male employees with gastrointestinal cancer had less quantitative job overload and a better physical environment, such as noise, lighting, temperature, and ventilation, than those without cancer [13]. Eye strain is the most common of visual display terminals (VDT)-related symptoms among workers [14], and the causes of lower back pain include not only diseases, but also behavioural factors (e.g., prolonged static sitting posture) and environmental factors (e.g., lighting, layout of work space and equipment) besides [15]. Therefore, it is possible that our findings of the low risk of eyestrain and lower back pain among male employees with gastrointestinal cancer may be due to consideration of the working environment. In Japan, support for cancer patients in the workplace is positioned as one of the important measures in the Basic Plan to Promote Cancer Control Programs, and “Guidelines for Supporting the Balance between Treatment and Working Life in the Workplace” have been presented [16]. However, consideration for cancer patients and systems differs depending on company size, and the smaller the number of employees, the less likely the company is to have introduced various systems [17]. Since this study population is employees of a large company, and the findings may differ for employees of small and medium-sized companies, further studies targeting employees of various-sized companies are needed.

Second, female employees with cervical cancer had higher ORs for heart diseases and hypertension than those without cancer (Figure 3). Although discussing the definite cause of the higher risks of hypertension and heart diseases is difficult, there is a possibility of side effects from bevacizumab, one of the molecular target drugs used in the drug therapy of advanced or recurrent cervical cancer. It has been reported that when bevacizumab is used in combination with chemotherapy, there is an increased risk of hypertension and thromboembolism [18], and that hypertension, neutrophil count decreased, and neutropenia are frequently observed [19]. Since this study did not investigate cancer treatment methods, further verification is required, but it is important for occupational health staff to understand the cancer treatment methods and the status of heart diseases and hypertension of female employees with cervical cancer.

Third, female employees with breast cancer had a higher OR for musculoskeletal diseases than those without cancer (Figure 4). Although they had a higher rate of hypertension and mental illness than those without cancer (Table 2), which was consistent with reports for hypertension on adults with cancer in the U.S. [7], for unipolar depression in female patients of breast cancer in Denmark [20], and for mood disorders in female patients with breast cancer in Taiwan [21], this difference disappeared after adjusting for age, job position, and department (Figure 4). Since it was reported that non-workers have a 2.6 times higher risk of depression than workers among Spanish female patients diagnosed and/or treated for breast cancer [22], it is possible that employment may reduce the risk of mental illness in female patients with breast cancer. Our finding for musculoskeletal diseases is consistent with that due to upper limb impairment, one-half of Finnish female patients who had undergone surgery for breast cancer experienced limitations in lifting, carrying, and reaching out one year postoperatively [23], and that 62% of American female employees with breast cancer who were one year after diagnosis had difficulty with heavy lifting [24]. Therefore, employers should ascertain the presence of musculoskeletal diseases in female employees with breast cancer and consider their job descriptions accordingly.

Fourth, for both sexes, employees with cancer did not differ from those without cancer regarding depressive symptoms and anxiety. This is partially consistent and partially inconsistent with previous studies, as follows. Regarding male employees with gastrointestinal cancer, our findings of depressive symptoms support those of Japanese workers with cancer [5] and of workers in four Nordic countries after primary treatment for cancer [6], whereas our finding of anxiety does not support those of workers after primary treatment for cancer [6]. Similarly, regarding female employees, our finding of anxiety in those who with cervical cancer was similar to those of French females who were 10 years post-cervical cancer diagnosis [25], whereas our findings of depressive symptoms and anxiety in those who with breast cancer were not similar with those of U.S. female workers who have completed treatment for breast cancer [4]. These may be due to the difference in the scales of measurement used and the target population, and further validation studies are needed.

This study has some limitations. First, the study population was large; however, the subjects were employees of one company and thus may have included occupational bias in these findings. Therefore, verifying these findings for employees in multiple occupations is needed. Second, since employees with cancer were extracted from health insurance claims data, details of cancer, such as cancer treatment method, duration from cancer diagnosis, or whether the cancer being treated was due to recurrence, were unknown. As these factors may also be associated with the health problems of employees with cancer, future studies should take these factors into account in more detail. Third, since stratified analysis by sex and cancer type was conducted in this study, only three cancer types were selected due to the limitation of sample size. Future studies on other cancer types are desirable.

Despite these limitations, to the best of our knowledge, this is the first study to examine various health problems, such as depressive symptoms, anxiety, indefinite complaints, and diseases other than cancer, among Japanese employees with cancer compared to those without cancer. The study findings suggest that the focus should be on female employees with cancer, especially the risks of heart diseases and hypertension in those with cervical cancer and musculoskeletal diseases in those with breast cancer. Therefore, employers should ensure that these employees' work does not affect the incidence or worsening of such diseases and take measures to continually balance treatment and work.

## Conclusions

This study examined 19 health problems, such as depressive symptoms, anxiety, indefinite complaints, and diseases in Japanese employees with cancer compared to those without cancer, stratified based on sex and cancer type, using data from the stress checks and health insurance claims. The results showed that male employees with gastrointestinal cancer experienced less eyestrain and lower back pain than those without cancer. Female employees with cervical cancer had more heart diseases and hypertension, whereas female employees with breast cancer had more musculoskeletal diseases than those without cancer. Therefore, workplace support measures for these health problems among female employees with cervical or breast cancer should be considered.
